# Effect and safety of Yiqi-Shengjin granules in hypertension management among patients with abnormal glucose metabolism: a study protocol for a randomized, double-blind, placebo-controlled clinical trial

**DOI:** 10.3389/fcvm.2026.1793094

**Published:** 2026-05-11

**Authors:** Haoyue Shi, Ruiqing Li, Juju Shang, Zhilin Chen, Xuerui Zhao, Sinai Li, Linjing Yang

**Affiliations:** 1 Beijing Hospital of Traditional Chinese Medicine, Capital Medical University, Beijing, China; 2 Graduate School, Beijing University of Chinese Medicine, Beijing, China; 3 Beijing Institute of Chinese Medicine, Beijing, China; 4 Capital Medical University, Beijing, China

**Keywords:** abnormal glucose metabolism, high blood pressure (HTN), hypertension, traditional Chinese medicine (TCM), Yiqi-Shengjin granules

## Abstract

**Background:**

The co-occurrence of hypertension and abnormal glucose metabolism—a spectrum encompassing prediabetes and diabetes—substantially elevates the risk of cardiovascular disease (CVD). Effective management of this high-risk clinical overlap remains a therapeutic challenge. Yiqi-Shengjin granules (YQSJ), a traditional Chinese herbal formulation, have been employed as a hospital preparation at Beijing Hospital of Traditional Chinese Medicine for over three decades, primarily for the management of glucose metabolism disorders, with an annual treatment cohort exceeding 1,000 patients. Although preliminary clinical observations suggest potential benefits of YQSJ in patients with hypertension complicated by diabetes, robust evidence from standardized clinical trials is lacking. Therefore, a well-controlled clinical study is warranted to systematically evaluate the efficacy and safety of YQSJ in this specific population.

**Methods/design:**

This will be a randomized, double-blind, placebo-controlled study. After being screened and providing signed informed consent, participants will be evenly assigned to experimental or control groups in a 1:1 ratio. The experimental group will receive YQSJ 3 g twice daily and losartan 50 mg once daily, whereas the control group will receive YQSJ placebo 3 g twice daily and losartan 50 mg once daily. The treatment period will last for a total of 8 weeks (56 days), with follow-up visits scheduled every 2 weeks. The primary endpoint will be the difference in drug efficiency between the two groups. The secondary endpoints include 24 h blood pressure indicators, glucose metabolism indicators, lipid metabolism indicators, vascular damage indicators, insulin resistance indicators, oxidative stress indicators, and major adverse cardiovascular events (MACE). Safety and efficacy outcomes will be assessed both before and after the 8-week medication period.

**Discussion:**

This study aims to provide clinical evidence on the efficacy and safety of YQSJ in the treatment of hypertension in patients with abnormal glucose metabolism. Furthermore, we will explore the potential mechanism through which YQSJ might exert hypotensive effects and adjust glucose metabolism by examining pertinent indicators of vascular and oxidative stress, as well as its influence on clinical symptoms, risk factors associated with hypertension, and the target organs affected by this condition.

**Clinical Trial Registration:**

identifier ChiCTR2200062453.

## Backgrounds

The co-occurrence of hypertension and abnormal glucose metabolism—encompassing prediabetes and diabetes—poses a significant and escalating global cardiovascular challenge (1, 2). This combination synergistically amplifies the risk of major adverse cardiovascular events, creating a complex therapeutic landscape (3, 4). While conventional antihypertensive agents remain foundational, their limited impact on glycemia and associated side effects underscore the need for complementary strategies that address both conditions simultaneously (5–7).

Traditional Chinese Medicine (TCM), with its holistic approach and multi-target mechanisms, offers a unique paradigm for managing complex conditions like hypertension combined with glucose metabolism disorders. Historical records in ancient texts such as the Huangdi Neijing describe syndromes related to these conditions, and modern clinical practice continues to utilize TCM formulations for their management (8, 9). For instance, clinical studies have demonstrated the efficacy of specific TCM preparations, such as Songling Xuemaikang Capsule for blood pressure reduction in hypertensive patients (10) and Xiaoke Decoction for improving glycemic control (11), underscoring the potential of TCM in this field.

Yiqi-Shengjin Granules (YQSJ) is a hospital-prepared TCM formulation (Approval No. Z20053315). It has been clinically applied for over 30 years at Beijing Hospital of Traditional Chinese Medicine, a leading national TCM institution whose cardiovascular department is recognized as a Key National TCM Specialty. YQSJ is annually administered to over 1,000 patients for conditions related to hypertension and glucose metabolism disorders (12). The formula comprises ten medicinal herbs, including Panacis Quinquefolii Radix, Astragali Radix, Scrophulariae Radix, Citri Sarcodactylis Fructus, Lycii Fructus, Dendrobii Caulis, Amomi Fructus, Ophiopogonis Radix, Polygonati Odorati Rhizoma, Polygonati Rhizoma (13). All of them are listed as safe for use in health foods by the National Health Commission of the People's Republic of China (https://www.nmpa.gov.cn/).

Preclinical and pharmacological research provides a scientific basis for its application. YQSJ has been shown to significantly reduce random blood glucose and the insulin resistance index in spontaneous type 2 diabetic KKAy mice (14). Bioactive components of YQSJ, such as Ginsenoside Rg1, Harpagoside, and Calycosin-7-O-β-D-glucoside, have been identified in patient serum following administration (13). Network pharmacology and molecular docking analyses suggest that its core mechanism may involve modulation of the IRS-1/PI3K/AKT signaling pathway (13), a key pathway in both glucose metabolism and cardiovascular function. Several individual components of YQSJ have also been independently reported to possess antihypertensive properties (15–17). Pharmacological evidence indicates that these herbal components may improve endothelial function, reduce oxidative stress, and enhance insulin sensitivity. Furthermore, the formula has been included in a regional medical innovation promotion database, and previous clinical trials have explored its efficacy in prediabetes (18), diabetic cardiomyopathy (19), and stable angina (20).

Despite its long-term clinical use and promising preliminary data, robust evidence from randomized controlled trials regarding the efficacy and safety of YQSJ specifically for hypertension in patients with abnormal glucose metabolism is lacking. To address this gap, we designed this randomized, double-blind, placebo-controlled clinical trial. The present article details the study protocol.

## Methods

### Objectives

This study aims to assess the clinical effects of YQSJ on blood pressure in patients with abnormal glucose metabolism and to provide a reliable experimental basis for the treatment of hypertension and impaired glucose metabolism.

### Design

This is an exploratory study with a randomized, double-blind, placebo-controlled design. All protocol modifications were made before participant enrollment to improve feasibility, ensure operational consistency, and comply with ethics requirements. They were planned and implemented by the study team, reviewed and approved by the ethics committee, as detailed in Supplementary Material.

After screening and voluntarily signing the informed consent form, 72 participants recruited from the Beijing Hospital of Traditional Chinese Medicine will be randomly and evenly assigned into the experimental and control groups on the basis of the randomized numerical table generated by SAS software.

**Figure 1:**
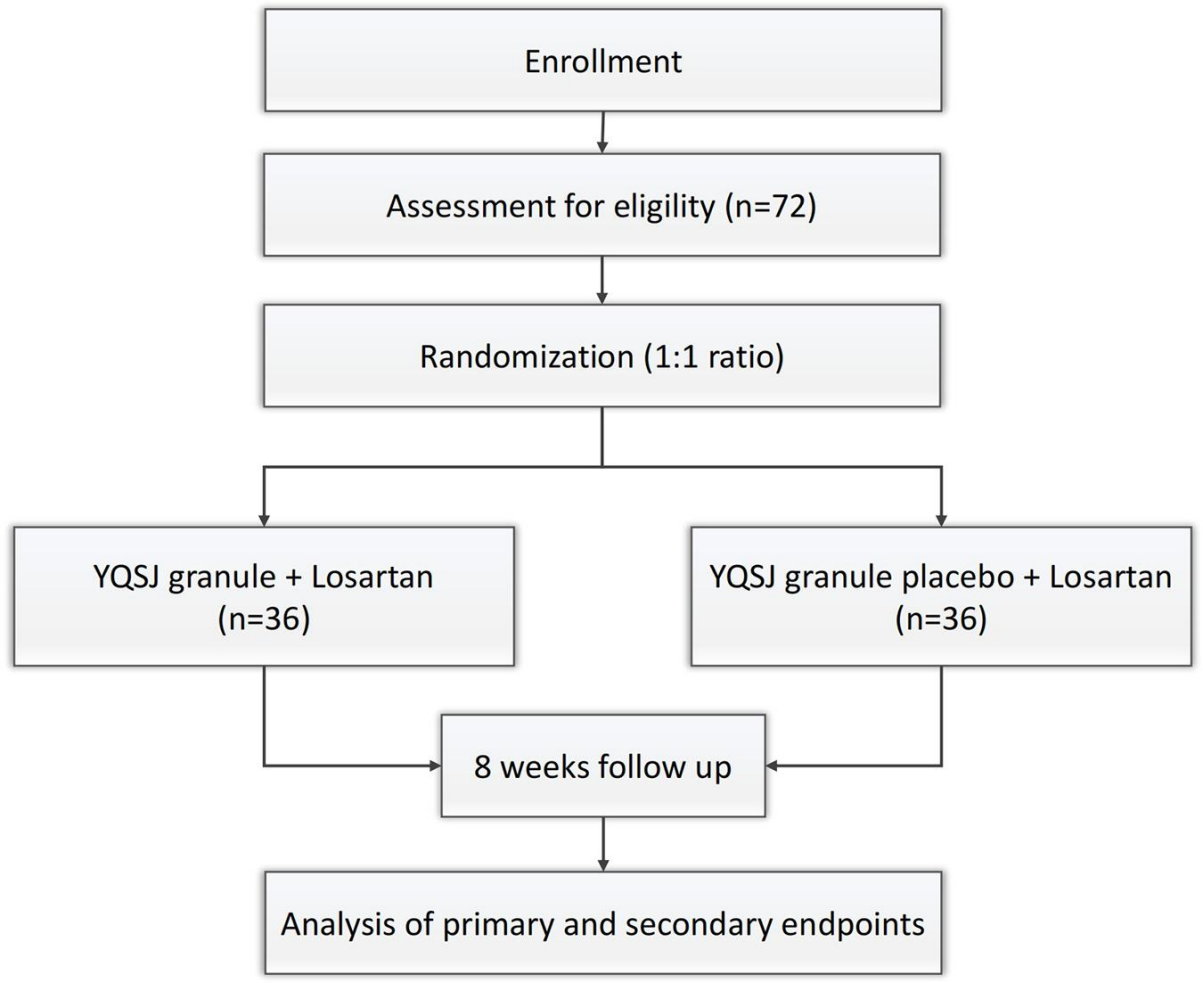
Flowchart of this study.

The experimental group will be treated with YQSJ 3 g twice a day and losartan 50 mg once a day, whereas the control group will be treated with YQSJ placebo 3 g twice a day and losartan 50 mg once a day. The patient's basic drug regimen remained unchanged. The treatment will last 8 weeks (56 days) in total, with follow-up visits every 2 weeks. Safety and efficacy indicators will be observed before and after 8 weeks of medication (Figure 1).

### Recruitment

Participants will recruit from Beijing Hospital of Traditional Chinese Medicine through notices in hospital and newspapers. Advertisement approved by the Research Ethics Committee will be used to promote enrollment. The principal investigator, together with a well-trained attending physician, will identify potential patients on the basis of the eligibility criteria. All the included participants were informed face to face of the entire study according to the Research Ethics Committee and voluntarily signed the informed consent form.

The eligibility criteria for the study participants were as follows:

Inclusion criteria: patients who met all of the following criteria.

1. Understand the process of this trial and provide informed consent.
2. Patients were diagnosed with impaired fasting glucose, abnormal glucose tolerance or type 2 diabetes mellitus in accordance with the ADA standard (3), and HbA1c < 8% within the past 2 weeks.
3. The patient was diagnosed with primary hypertension (grade 1–2) in accordance with the AHA standard (1). It is defined as systolic blood pressure (SBP) of 140–179 mmHg and/or diastolic blood pressure (DBP) of 90–109 mmHg.
4. Male aged between 18 and 75 years.

Exclusion criteria: Patients who met any of the following criteria.

1. Severe infection, trauma and/or recent or planned surgery.
2. Severe conscious impairment, severe cognitive dysfunction, or severe psychiatric disorders;
3. Those with other comorbidities, such as chronic kidney disease and liver disease (hepatic inadequacy: alanine amino transferase or alkaline phosphatase levels more than twice the upper normal limit). Renal inadequacy: serum creatinine level ≥133 mmol/L)

Withdrawal criteria: Patients who met any of the following criteria.

1. Discrepant the eligibility criteria after inclusion.
2. The participants were asked to withdraw.
3. Allergic reactions, serious adverse reactions, adverse events, or intolerance
4. Unable to perform the test as needed (poor compliance).

### Ethics

This protocol was approved by the clinical research ethics committee of the Beijing Hospital of Traditional Chinese Medicine (2022BL02-028-03), where the study takes place. It has been registered with the Chinese Clinical Trial Registry (ChiCTR2200062453), which is listed in the WHO Registry Network. The trial complies with the principles of the Declaration of Helsinki and the principles of good clinical practice.

### Randomization and blinding

All the enrolled patients were included in the clinical trial according to the order of random table numbers from the numerical table generated by SAS software. The ratio of the experimental group to the control group is 1:1.

For blinding, the allocation code for each participant will be placed in opaque envelopes. The envelopes also contain details of the treatment options, possible adverse events, and emergency measures. The envelopes and treatment plans of each participant will not be accessible to all the researchers or participants. The drug and the placebo are similar, and the manufacturer will label the random codes on the package according to the principles of GCP.

Each coded experimental drug is accompanied by a corresponding emergency letter containing a slip of paper indicating the type of the coded drug to be used in the event of an emergency to break the blind. The emergency letters were sealed and sent to each clinical trial center with the corresponding numbered test drug, which is responsible for keeping it and should not be opened unless necessary. It should be opened by the investigator in the event of an emergency, or if the subject requires resuscitation, it is necessary to know what treatment the subject is receiving. Once opened, the numbered subject will be withdrawn from the trial and treated as a shedding case, and the investigator will record the reason for withdrawal on the case report form. All contingency letters will be retrieved with the case report form at the end of the trial for blinded review at the end of the trial.

### Intervention

YQSJ (production batch number 181201) and YQSJ placebo (production batch number 181202) were produced and packed in a single batch by the Beijing Institute of Traditional Chinese Medicine. According to the preparation process, 1 g of the finished granules corresponds to approximately 1.67 g of crude herbal materials. Each bag of YQSJ contained 5 g of crude drug. The dosage is consistent with the recommended dosage in previous pharmacology studies and clinical practice (13). As tested, the drug conformed with the quality specified in the Chinese medicine standards published by the State Food and Drug Administration. Phytochemical analysis via LC-MS performed by Beijing Hexin Technology Co., Ltd. was used to identify the constituent compounds. The total ion chromatogram of Liquid chromatography-mass spectrometry, plant sources and compounds were presented in Supplementary Figures S1–S2 and Table S1–S2). Losartan will be purchased from the Pharmaceutical Branch of China Pharmaceutical Holdings Beijing Co., Ltd. (Unified Social Credit Identifier 91110101101297579G).

The experimental group will be treated with YQSJ 3 g twice a day and losartan 50 mg once a day, whereas the control group will be treated with YQSJ placebo 3 g twice a day and losartan 50 mg once a day. The patient's basic drug regimen remained unchanged. The treatment will last 8 weeks (56 days) in total, with follow-up visits every 2 weeks. Safety and efficacy indicators will be observed before and after 8 weeks of medication.

All participants will receive losartan as the background antihypertensive therapy throughout the study period. The use of other antihypertensive medications will be prohibited unless clinically necessary. If additional antihypertensive treatment is required due to uncontrolled blood pressure or safety concerns, the participant will be managed according to clinical judgment and may be withdrawn from the study if necessary. Medication adherence will be monitored through regular follow-up visits, drug accountability records, and patient self-reports to ensure compliance with the study protocol.

To minimize the impact of potential confounding factors, all participants will receive standardized lifestyle recommendations, including diet and physical activity guidance, based on current hypertension management guidelines. Participants will be instructed to maintain stable lifestyle habits during the study period and to avoid significant changes in diet, exercise, or weight. Body weight will be recorded at baseline and monitored during follow-up visits. Any substantial changes will be documented and considered in the analysis.

### Endpoint measurements

#### Primary endpoint

The primary endpoint is the difference in drug efficiency between the two groups. Efficiency is defined as follows: blood pressure is reduced to a threshold set out in the Guiding Principles for Clinical Research of New Chinese Medicines (21). The level of glycosylated hemoglobin is decreased by at least 2% (22).

#### Secondary endpoints

1. 24-h blood pressure indicators: average SBP, DBP, mean arterial pressure (MAP), blood pressure variability and circadian blood pressure amplitude
2. Glucose metabolism indicators: fasting blood glucose, 2 h postprandial blood glucose, glycosylated hemoglobin, CMI = WHtR × [TG (mmol/L)/HDL-C (mmol/L), WHtR = waist circumference (cm)/height (cm) (23), HOMA-IR = FBG (mmol/L) × FINS (μU/mL)/22.5 (24)
3. Lipid metabolism indicators: triglycerides, cholesterol, LDL, HDL
4. Vascular damage indicators: vascular endothelial function markers, brachial and ankle artery pulse wave velocity, ankle-brachial index
5. The oxidative stress indicators: superoxide dismutase (SOD) and malondialdehyde (MDA).
6. Major adverse cardiovascular events (MACEs) and disease regression within 6 months of treatment initiation
7. Safety indicators: Hepatic and renal function, routine blood results and routine urine results will be assessed at baseline and at the treatment endpoint.

### Laboratory tests

Blood samples will be collected and centrifuged within 10 min. The supernatant fluid will be pipetted and stored in a cryotube at −80 °C in Beijing Hospital of Traditional Chinese Medicine, Capital Medical University. The levels of hepatic and renal function indicators, routine blood and urine tests will be assessed at the Clinical Laboratory in the same hospital. The cryopreserved supernatant fluid will be tested via an enzyme-linked immunosorbent assay (ELISA) kit or chemical kit within 6 months to assess the serum levels of SOD and MDA. The instruments used during testing include an electronic balance (YP30001, Shanghai Guangzheng Medical Instruments Co., Ltd., China), a mini-shaker [MS1 Minishaker, IKA (Guangzhou) Instruments and Equipment Co., Ltd., China], a desktop low-speed automatic-balancing centrifuge (LDZ5- 2, Beijing Jingli Centrifuge Co., Ltd., China), an electric constant-temperature water tank (HW. W21.600, Beijing Changfeng Instrument and Meter Company, China), and a multifunction microplate reader [SpectraMax M2, Meigu Molecular (Shanghai) Instruments Co., Ltd., China].

### Data collection and management

**Figure 2:**
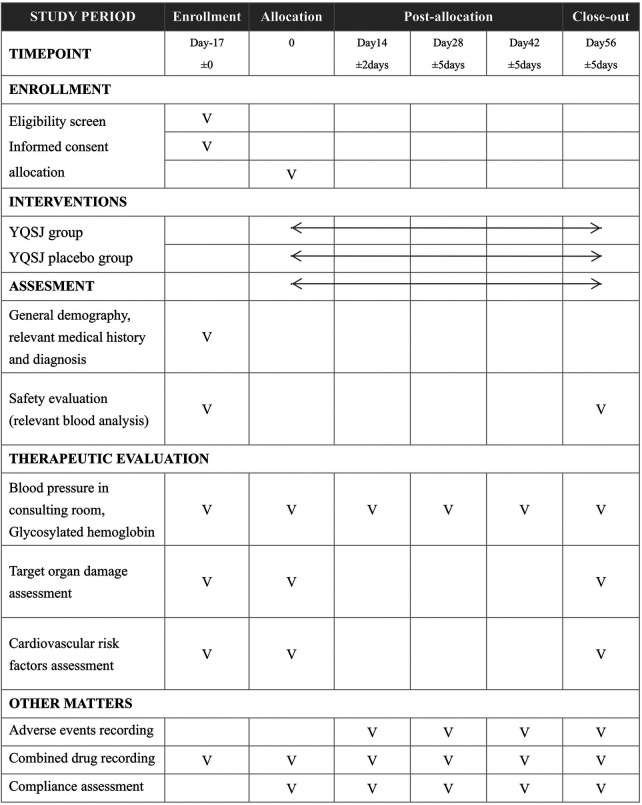
Schedule of enrollment, interventions, and assessments.

Data for each participant will be recorded in a case report form. Patients are required to attend the assessment every 14 days and finish the trial with 5 evaluations, including the enrollment evaluation (0 days, 14 ± 2 days, 28 ± 5 days, 42 ± 5 days, and 56 ± 5 days). Each evaluation included general demography, relevant medical history and diagnosis, safety evaluation, blood pressure assessment in the consultation room, symptom improvement assessment, target organ damage assessment, cardiovascular risk factor assessment, and other methods, such as adverse event recording, combined drug recording, and compliance assessment, as shown in Figure 2. Baseline blood pressure will be measured using standardized procedures, and the average of at least two measurements will be recorded for each participant.

Data management will be performed by two researchers through a website completely independent of the investigators and the sponsor. The system, which will be protected by passwords, will be managed by Beijing Cardiar Technology Co., Ltd. (Unified Social Credit Identifier 91110 10,655 68,170 240). All data supporting the conclusions of this trial will be stored in this system. To ensure data integrity and ease of storage, we use data rules, valid values, and scope checks. The system reports missing data and data errors. The data may be changed, but all changes will be tracked. The trial will be audited by Beijing Inruida Pharmaceutical Technology Co., Ltd. (Unified Social Credit Identifier 91110105MA00H1U54L). These data management procedures will be undertaken completely independently of the investigators and the sponsor. All the data management, including coding, storage, security, and cleaning, will be performed according to the data rules, valid values, and scope checks. The conduct of the trial will be audited through a website (http://www.cardiar.com/zryygxy). The personal information of all participants will be fully protected. The original case report forms will be kept for 5 years after the end of the trial.

### Adverse events

The study is largely a low-risk exploratory trial. However, any signs of adverse event symptoms that occurred during the observation period were required to inform the study nurse immediately and be recorded in the case report. Common adverse events (AEs) include gastrointestinal reactions such as malignant, abdominal distension, abdominal pain, skin reactions such as facial flushing, skin rash and mild cough. AEs should be reported to the research leader, sponsor, and ethics committee within 24 h by the clinical researcher for further treatment decisions. An AE will be categorized as related (definitely, probably, or possibly) or not related (unlikely or not related) to the study drug. An adverse drug reaction (ADR) will be defined as an AE that is considered related to the study drug.

### Statistical analysis

#### Sample size calculation

The sample size calculation was based on clinical research literature and preliminary clinical data. On the basis of the design of the superiority test, we assume that the efficiency of the placebo group is approximately 30%, and the efficiency of the experimental group is approximately 65%. In accordance with the statistical superiority test (superiority threshold of 0), *α* takes a unilateral value of 0.025. When the grouping ratio was 1:1 under the condition of 80% certainty, the control group and the experimental group needed 27 cases each as the evaluable number of cases. Considering the length of the district group and the 20% shedding rate, 36 patients were planned to be enrolled in each group, and a total of 72 patients were needed. PASS11 software was used to calculate the sample size.

#### Data analysis

SPSS 15.0 (PN: 32119001, SN: 5045602) will be used for statistical analysis. The measurement data are expressed as the means ± standard deviations. Between-group comparisons of two groups of normally distributed and variance-aligned continuous variables were performed via t tests. Comparisons between groups of two normally distributed continuous variables with chi-square variance between each stratum will be performed via analysis of variance (ANOVA) with randomized area group design data, and two-by-two comparisons between multiple groups will be performed via the SNK-q test. Comparisons between groups of nonnormally distributed continuous variables will be performed via the rank sum test. Comparisons between groups of count data were performed via the chi-square test, and Fisher's exact probability test will be used when the expected frequency is <5. Major adverse cardiac events will be evaluated via Cox regression analysis. *P* < 0.05 is considered a statistically significant difference. Baseline blood pressure levels will be included as covariates in the statistical analysis to minimize potential confounding effects.

## Discussion

The coexistence of hyperglycemia and hypertension represents a critical synergistic risk factor for CVD, substantially elevating both morbidity and mortality (25). Early and effective intervention in this high-risk population is therefore essential to attenuate the progression of atherosclerotic cardiovascular disease (ASCVD), improve long-term outcomes, and alleviate the growing burden of chronic disease management (26). Despite the clear clinical significance, there remains a notable gap in robust therapeutic strategies specifically targeting the confluence of glucose metabolism disorders and hypertension (27).

YQSJ represents a traditional Chinese medicine formulation with over three decades of clinical application in the management of metabolic disorders at a leading tertiary hospital. Preclinical pharmacological investigations suggest that its bioactive components may exert hypoglycemic and antihypertensive effects through modulation of the IRS-1/PI3K/AKT signaling pathway, a key regulator of metabolic and vascular homeostasis (13).

While prior research has primarily examined the efficacy of YQSJ in prediabetic populations, high-quality clinical evidence regarding its utility in patients with concurrent hypertension and abnormal glucose metabolism is lacking. This protocol outlines a randomized, double-blind, placebo-controlled trial designed to evaluate the blood pressure-regulating effect of YQSJ in this specific patient cohort. Beyond assessing conventional endpoints such as blood pressure and glycemic parameters, this study will incorporate a comprehensive evaluation of cardiovascular risk profiles, including lipid metrics, the triglyceride-glucose index, vascular function, and patient-reported health outcomes. An embedded health-economic analysis will further assess the cost-effectiveness of the intervention.

Looking ahead, planned multi-omics analyses of collected biological samples aim to elucidate the systemic mechanisms underlying the therapeutic effects of YQSJ, bridging clinical observations with molecular insights. We anticipate that this trial will contribute empirical evidence supporting the integration of TCM-based approaches into outpatient management pathways for this high-risk population.

## Limitations

This study acknowledges several limitations. The exclusion of female participants may affect the generalizability of the findings to broader populations. The pilot-scale sample size, while appropriate for an initial controlled investigation, may limit the statistical power to detect subtle treatment effects or fully capture population heterogeneity. Furthermore, the relatively short duration of follow-up restricts our ability to evaluate the long-term sustainability of treatment effects and clinical outcomes. Future studies with larger, more diverse cohorts and extended observation periods will be valuable in confirming and extending these preliminary findings.

## Trial status

This protocol (version 1.2) was approved by the clinical research ethics committee of the Beijing Hospital of Traditional Chinese Medicine (2022BL02-028-03), Beijing, China. The study started on 1st January 2024. Currently, 40 patients have been recruited. The study will be finished by June 2026.
