# Supplementary material for: Effect and safety of Yiqi-Shengjin granules in hypertension management among patients with abnormal glucose metabolism: a study protocol for a randomized, double-blind, placebo-controlled clinical trial
Source: Front Cardiovasc Med. 2026 May 11;13:1793094. doi: 10.3389/fcvm.2026.1793094 (PMC13199290; doi:10.3389/fcvm.2026.1793094)
Supplement: Supplementary file 2 [file Datasheet1.docx]

**
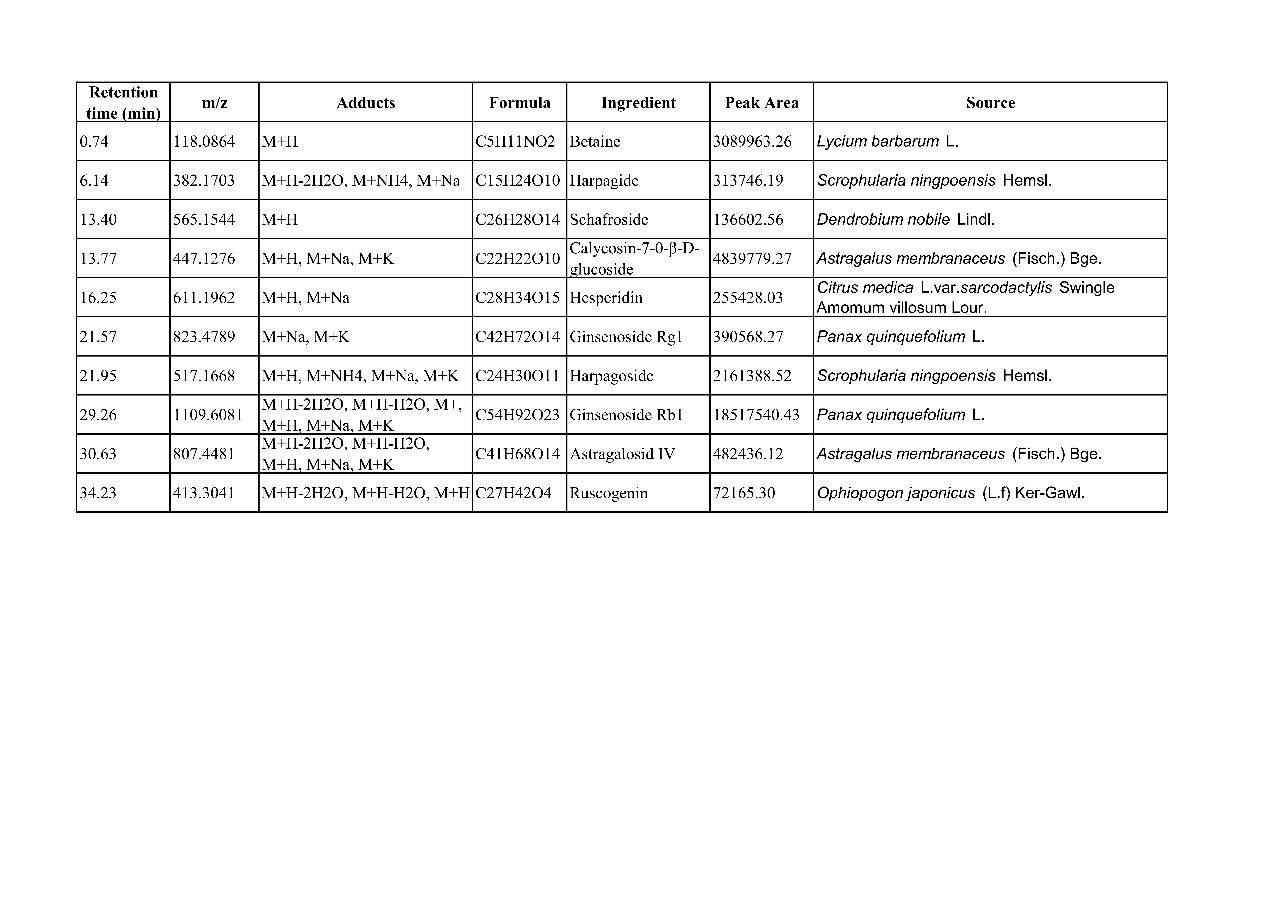
**

**Table S1 Compounds** **from UPLC-MS/MS analysis of Yiqi-Shengjin granules and their resources in positive mode**

^*^ Compared with reference compounds


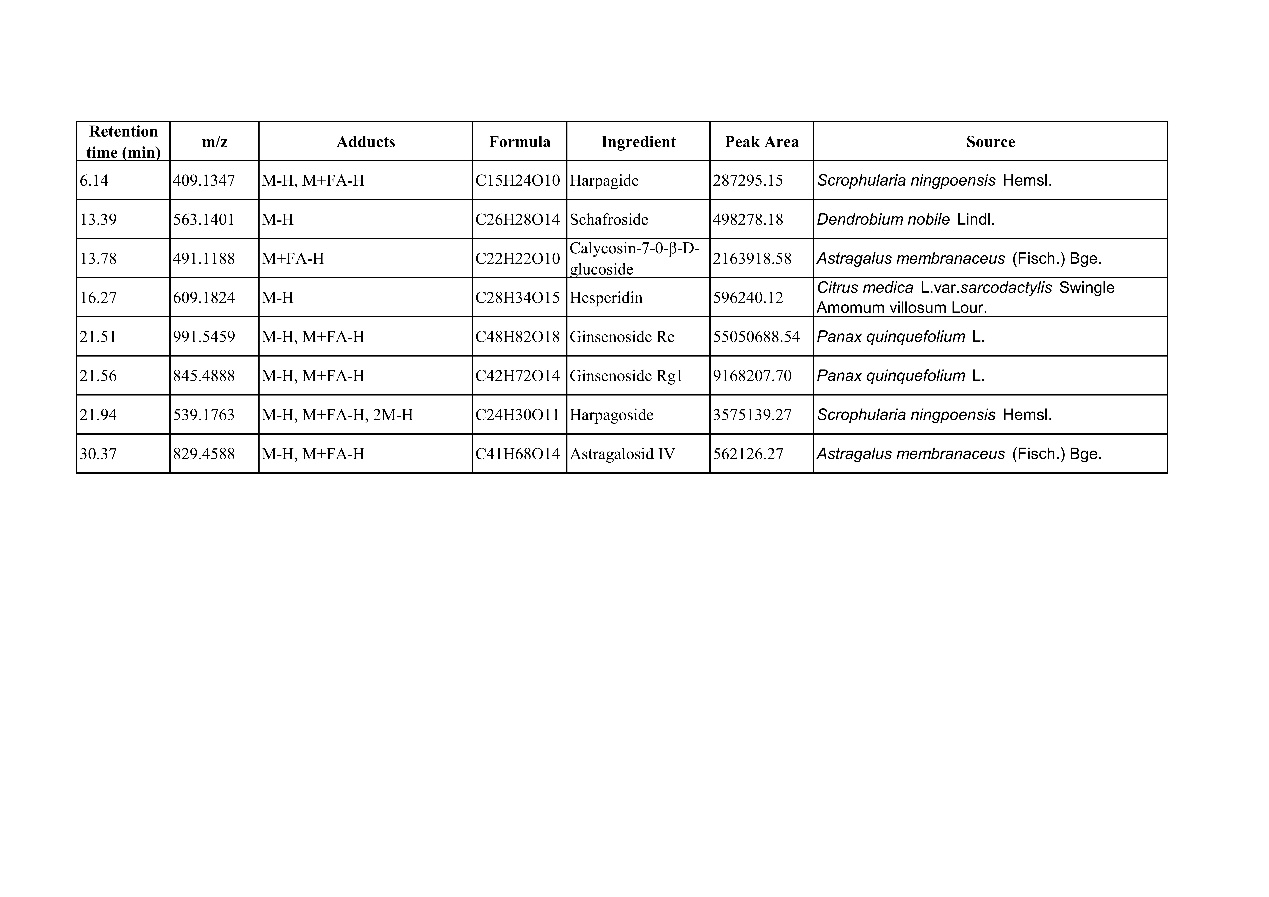


**Table S2 Compounds from UPLC-MS/MS analysis of Yiqi-Shengjin granules and their resources in negative mode**

^*^ Compared with reference compounds


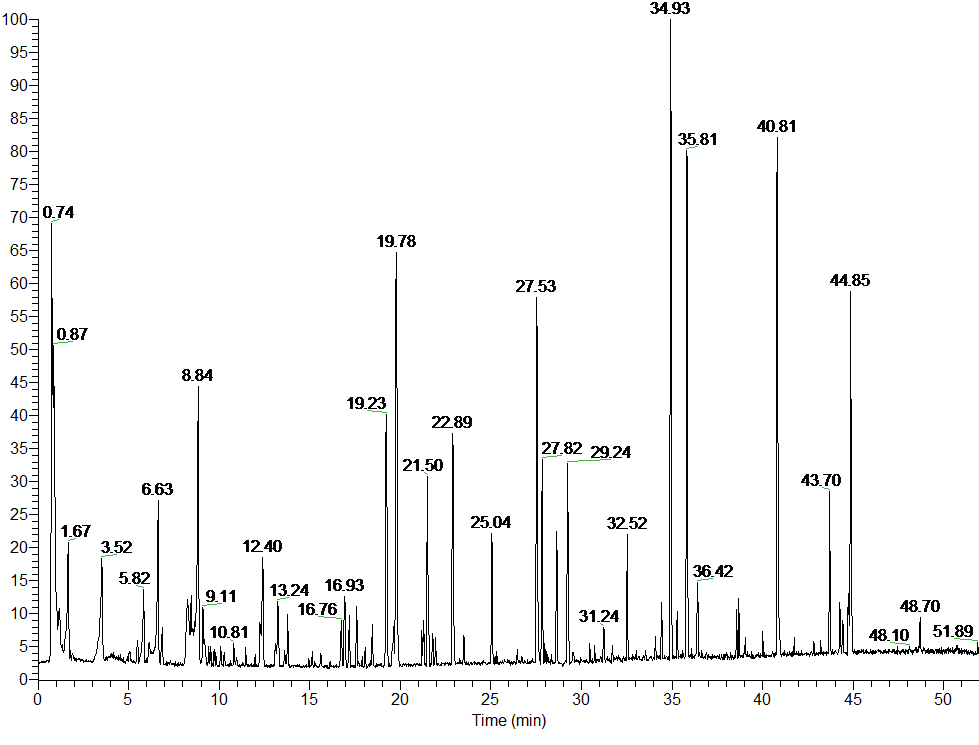


**Figure S1 The base peak chromatogram of Yiqi-Shengjin granules in positive mode.**


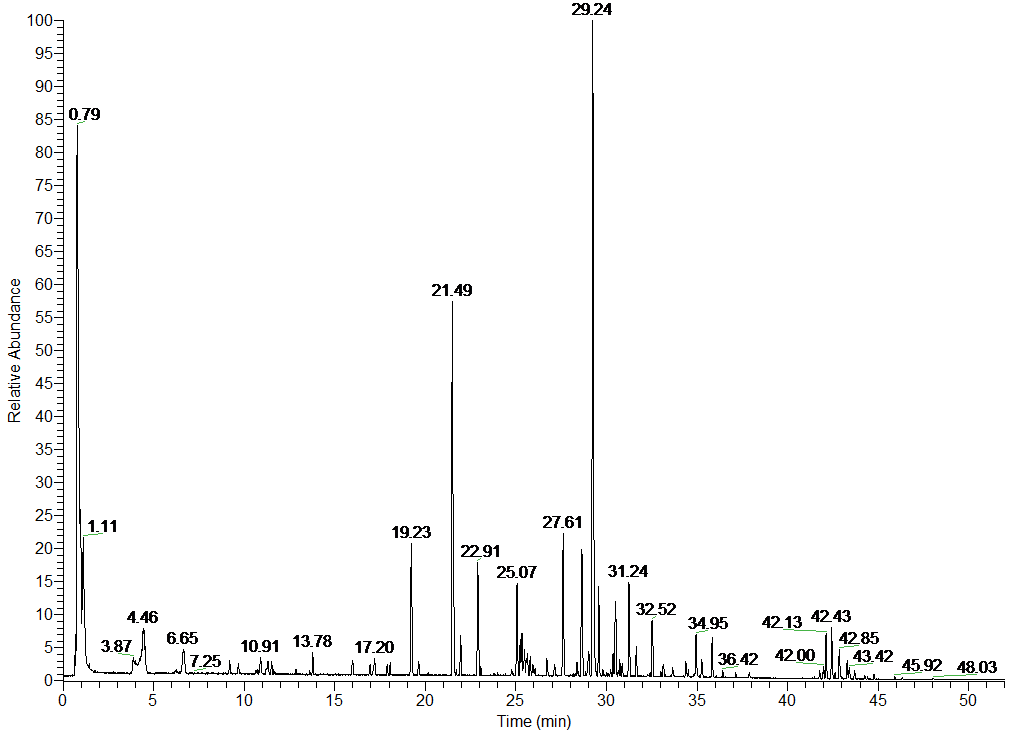


**Figure S2 The base peak chromatogram of Yiqi-Shengjin granules in negative mode.**
